# Spatiotemporal Characteristics of Health Collaboration Development in China’s Urban Agglomerations: An Empirical Analysis of Four Major Regions

**DOI:** 10.3389/ijph.2026.1608932

**Published:** 2026-04-09

**Authors:** Xiangfei Li, Wentao Zhu, Xumin Zhu

**Affiliations:** 1 School of Economics and Management, Tiangong University, Tianjin, China; 2 School of Public Administration and Policy, Renmin University of China, Beijing, China

**Keywords:** collaborative governance, public health, regional integration, urban agglomerations, urban network structure

## Abstract

**Objective:**

This study analyzed China’s four most politically significant and economically dynamic urban agglomerations—Beijing-Tianjin-Hebei (BTH), Yangtze River Delta (YRD), Pearl River Delta (PRD), and Chengdu-Chongqing (CY)—to explore their health development trends from 2005 to 2023 and compare their spatiotemporal characteristics in coordinated health development.

**Methods:**

We employed an integrated index construction method and an improved urban gravity model to build urban health network models, investigating the coordinated relationships and features of health development in these agglomerations.

**Results:**

Over 17 years, the four agglomerations showed both commonalities and unique differences in health coordination. The degree of coordinated development strengthened significantly, although it was notably influenced by major policies and public health events. Core cities exhibited substantial radiating effects on regional health coordination. PRD and YRD exhibited more pronounced growth in coordination intensity than BTH and CY. Distinct coordination patterns emerged: BTH displayed a unipolar radiation structure, YRD a polycentric network, PRD a core-periphery structure, and CY a dual-core, policy-driven coordination structure.

**Conclusion:**

The findings reveal the critical role of core cities and policy interventions in shaping regional health collaboration networks, providing insights into how to achieve more balanced health resource allocation and equity in urban agglomerations.

## Introduction

Within urban clusters, core cities often concentrate high-quality public health and medical resources, not only meeting local demands but also serving as regional hubs for resource distribution. The cross-regional flow and functional spillover of these resources are key indicators of enhanced collaborative health security capabilities within urban clusters. Rational allocation of resources within a region significantly improves the accessibility of medical and health resources across the entire area. Thus, it is necessary to further consider regional medical resource security capabilities from a holistic perspective under urban collaboration [1]. In these urban regions, medical resources are highly networked and coordinated among cities [2]. Core cities in these areas hold overwhelming advantages in terms of policy, talent, economic strength, and medical standards, benefiting not only the core cities themselves but also neighboring cities. For example, of China’s 125 national regional medical center construction projects, Beijing and Shanghai have exported 50 and 20 projects, respectively, to surrounding cities [3], achieving a certain degree of radiation effect for high-quality medical services. However, public health services within urban clusters still exhibit structural imbalances. On one hand, core cities possess significant advantages in policy, talent, financial investment, and medical standards; on the other hand, non-core cities and peripheral regions face substantial gaps in resource supply, health facilities, emergency response capabilities, and more. Inter-regional coordination mechanisms remain underdeveloped, forming an asymmetric “single-core-multi-periphery” structure.

Existing research has found that, in the context of cross-departmental and cross-regional collaboration, ambiguous responsibilities and an absence of performance evaluations lead to inefficiencies in coordination. For example, the imbalanced development of urban functions in China (emphasizing the economy and infrastructure while neglecting social services) has resulted in a gap in public health [4]. China’s grassroots emergency response capabilities in its medical and public health systems are weak [5]. In Beijing, the accessibility of secondary and tertiary hospitals for low-income communities is only one-fourth that of high-income communities, and the time required to reach primary healthcare institutions is twice as long for low-income groups [6]. Moreover, primary healthcare institutions provide limited medical services. Shanghai’s central urban areas also have insufficient coverage of walkable primary healthcare services, with 22.8% of communities experiencing resource shortages. Older adults and low-income families face exacerbated difficulties accessing healthcare due to socioeconomic barriers [7, 8]. A significant portion of healthcare resources is concentrated in developed regions, such as Beijing and Zhejiang, leading to resource surpluses, while areas such as Anhui and Jiangxi have a Gini coefficient as high as 0.88 when measured by service area [9].

Overall, although urban agglomeration integration has promoted resource consolidation and institutional coordination, significant imbalances and fragmentation persist in public health services. Existing literature primarily focuses on the macro-level structure of urban agglomerations, with insufficient comparative analysis of different types of cities within them, especially non-core cities. There is a lack of systematic research on the coordination mechanisms of non-core cities. This study adopts a micro-level perspective, focusing on the role differences of various cities in coordinated health development, aiming to provide theoretical support and empirical analysis to achieve balanced resource allocation and health equity within urban agglomerations.

At the regional level, the collaborative development of public health is not a one-dimensional “expansion of quantity” but rather a multidimensional, networked evolutionary process influenced by various internal and external factors. Different elements interact and couple to drive the deepening of cross-regional public health partnerships and the integration of service systems, thereby forming a more cohesive and dynamically evolving regional health collaboration network.

In recent years, studies have thoroughly revealed the multidimensional factors influencing the spatiotemporal evolution of public health collaboration in urban agglomerations and regions, particularly highlighting the critical role of transportation, technology, and policy interventions in resource flows and functional spillovers. Table 1 summarizes these influencing factors.

**TABLE 1:** Multidimensional factors affecting the spatiotemporal evolution of urban agglomerations or regional public health coordination (Beijing-Tianjin-Hebei, Yangtze River Delta, Pearl River Delta region, and Chengdu-Chongqing region, China, 2023).

Category	Authors and year	Research topic
Transportation	Wang and Nie [10]	The association between transportation infrastructure and health
	Dalen [11]	Changes in the number of Americans seeking medical treatment abroad (2007–2017)
	Zhang et al. [12]	Association between traffic density, population mobility, and confirmed COVID-19 cases
	Li et al. [13]	Spatiotemporal evolution of COVID-19 in China and its correlation with transportation networks
Technology	Zhao et al. [14]	The impact of the digital economy on public health efficiency
	Yang [15]	Differences in internet usage frequency and health levels between urban and rural areas
	Haft and Allen [16]	Effectiveness of the health information exchange platform CRISP
Policy intervention	Rangachari et al. [4]	The U.S. Affordable Care Act and social determinants of health (SDOH) interventions
	Zhao et al. [17]	The impact of China’s low-carbon city pilot program on the structure of the healthcare industry
	Jiao and Zhang [18]	Cross-regional collaborative governance of Japan’s nuclear contaminated water incident
	Guo et al. [19]	Effectiveness of Beijing-Tianjin-Hebei healthcare collaborative development policies
	Butorina and Borko [20]	Functional positioning and collaborative mechanisms in regional integration
	Toma and Laurens [21]	Regional coordination experience of European integration

In recent years, spatial measurement technology has achieved significant progress both theoretically and practically as a crucial tool for revealing the patterns of public health collaborative development.

Spatial measurement, as a crucial analytical tool, has been widely applied in recent years in the field of public health. For example, Deng et al. studied the Guangdong-Hong Kong-Macao Greater Bay Area, using the space-time cube (STC) model and emerging hot spot analysis to confirm the stress effect of high-temperature environments on public health security, emphasizing the need to embed heat island mitigation strategies in urban planning to reduce population health risks [22]. Li employed the Dagum Gini coefficient and spatial econometric models to analyze medical service supply across 31 Chinese provinces from 2012 to 2020, revealing regional disparities characterized by “convergence in the east and west, lagging in the central region” [23]; Tang and Tan’s four-dimensional indicator system found that factors such as soil erosion control and population density directly influence the risk resilience of public health systems [24]; spatiotemporal evolution models play a key role in simulating epidemic spread and allocating medical resources. For instance, case distribution predictions based on kernel density estimation can improve emergency supply delivery efficiency by 35%, while spatial autocorrelation analysis highlights the need to strengthen vaccination coverage in key areas [13].

Compared to existing research, the main contributions of this study are as follows:

1. Focusing on China’s four most dynamic urban agglomerations, this study constructs a spatial gravity structure among cities in the field of public health, exploring for the first time the characteristics of collaborative healthcare development in China’s major urban agglomerations from a spatiotemporal perspective.
2. Fully considering China’s political characteristics, the study examines the leading role of major events, key policies, and politically influential core cities in regional healthcare collaboration.
3. The study also investigates the role of non-core cities in healthcare collaboration, focusing on internal structural differences within urban agglomerations, and reveals the spatiotemporal network characteristics exhibited during the collaborative development process of China’s four major urban agglomerations.

## Methods

### Study Area

This study covers health data from four key regions: the Beijing-Tianjin-Hebei urban agglomeration (BTH), the Yangtze River Delta urban agglomeration (YRD), the Pearl River Delta urban agglomeration (PRD), and the Chengdu-Chongqing metropolitan area (CY).

The most economically developed prefecture-level cities within each urban agglomeration were selected for this research, including 13 cities in the Beijing-Tianjin-Hebei urban agglomeration, 26 cities in the Yangtze River Delta urban agglomeration, 9 cities in the Pearl River Delta urban agglomeration, and 16 cities in the Chengdu-Chongqing urban agglomeration, totaling 64 cities. According to the official administrative hierarchy standards for Chinese cities, the cities in the study area are categorized accordingly [25, 26].

### Constructing a Healthy Urban Network Model

This study attempts to build a network model between cities based on their different urban health development levels to analyze their coordinated development.

First, we constructed an Urban Health Index (UHI)that represents the health development level of each city to analyze the developmental changes of different cities from 2005 to 2023.

Next, the study analyzed the synergy level of health development among different cities within urban agglomerations and constructed a Regional Health Collaborative Development Index (HCDI) to reflect the internal synergy level of the urban agglomerations.

On this basis, the synergy consistency of health development levels among different cities was analyzed using an improved urban gravity model.

Finally, an in-depth analysis was conducted of the constructed urban network model, extracting and summarizing four structural characteristics of the spatiotemporal collaborative development of urban agglomeration health.

This study, referencing Evangelista et al. [27], Korir [28], and Al-Ghamdi et al. [29], selected representative health development indicators. These include metrics reflecting urban health development levels, such as the quantity of health resources, health status, and local economic development levels. Additionally, indicators influencing inter-city synergy are chosen, including distance between cities, travel time, and policy support for collaborative development, as shown in Table 2.

**TABLE 2:** Required indicators for this study (Beijing-Tianjin-Hebei, Yangtze River Delta, Pearl River Delta region, and Chengdu-Chongqing region, China, 2023).

Dimensions	Subordinate indicators	Attribute
Health resources	Number of hospitals	+
	Number of practicing physicians	+
	Number of hospital beds	+
Health expenditure	Per capita total health expenditure (yuan)	-
Social development status	GDP	+
	Transportation distance between cities	-
	Transportation time between cities	-
Policy support intensity	Number of coordinated development policies issued	+

This study primarily utilized data sourced from the *China Health Statistical Yearbook* and the *China City Statistical Yearbook,* along with statistical yearbooks and publicly available data from individual cities and their respective provinces; policy documents were mainly obtained from the official websites of local Health Commissions and Medical Security Bureaus. The health-related policies examined in this study refer to government-issued policy documents closely related to coordinated health development. The selection criteria for the policy samples were as follows: first, ensuring that the policy text content was closely related to health, with systematic searches conducted using keywords such as “health coordination” “medical collaboration” and “healthcare cooperation”; second, government-issued laws, regulations, and measures were filtered, primarily selecting local normative documents while excluding those irrelevant to the research objectives.

Policy Quantification Method: The “Policy Support Intensity” indicator was quantified through a systematic content analysis of relevant government documents. The process involved three steps: (1) Document Collection: Policy documents were retrieved from the official websites of local Health Commissions and Medical Security Bureaus, using keywords such as “health coordination,” “medical collaboration,” and “healthcare cooperation.” (2) Screening and Classification: Collected documents were filtered to include only those containing substantive measures for promoting regional health collaboration. Each policy was then classified by its administrative level (national, provincial, or municipal). (3) Scoring: A scoring system was applied, wherein each policy received a base score of 1. This score was then weighted by its administrative level (national = 3, provincial = 2, municipal = 1) and by the specificity of its collaborative measures (detailed implementation rules = 2, general guidance = 1). A city’s annual “Policy Support Intensity” was the sum of the weighted scores of all relevant policies issued that year. This method ensured a more nuanced measurement than a simple policy count.

#### Urban Health Level Measurement

To better assess urban health development levels in this study, we constructed a UHI based on the indicator data in Table 2. Following the method by Sun et al. [30], positive and negative indicators were standardized separately to obtain weights. After determining the weights $X_{ij}$ , the UHI for each city was then calculated. For ease of subsequent computation, ${UHI}_{i}$ represents the final result as follows:

$$UHI_{i}=\sum_{j=1}^{m}W_{j}\cdot X_{ij}$$ (1)

#### Regional Health Collaborative Development Index

Based on previous calculations and analyses of various indicators, and considering their impact on regional health collaboration, the Analytic Hierarchy Process (AHP) method was used to score each indicator and construct a judgment matrix [31]. Based on this, the HCDI for each region from 2014 to 2023 was derived:

$$HCDI=\sum_{i=1}^{n}\left\{\left(\sum_{j=1}^{n}{x_{ij}}^{\prime }w_{j}\right)\left(\frac{\sqrt[{n}]{\prod_{j=1}^{n}a_{ij}}}{\sum_{k=1}^{n}\sqrt[{n}]{\prod_{j=1}^{n}a_{kj}}}\right)\alpha_{i}{\left[\sum_{l=1}^{n}\left(\frac{\sqrt[{n}]{\prod_{j=1}^{n}a_{lj}}}{\sum_{k=1}^{n}\sqrt[{n}]{\prod_{j=1}^{n}a_{kj}}}\right)\alpha_{l}\right]}^{-1}\right\}$$ (2)

In the equation, $x_{ij}$ represents the i-th region’s j-th standardized indicator value, $w_{j}$ is the j-th indicator’s weight for each year, n = 7 represents the number of indicators involved in calculating UHI for each city; $a_{ij}$ is the element in the i-th row and j-th column of the AHP judgment matrix A, used to calculate weights, and $\alpha_{i}$ represents the coefficient related to the i-th indicator.

#### Construction of an Urban Gravity Model Based on Health

Based on Newton’s law of gravitation and complex network theory, an urban gravity model for health was constructed using the formula:

$$G_{ij}=c\cdot \frac{\sqrt{{UHI}_{i}{gUH}_{j}}}{d_{ij}^{\gamma }}\cdot \exp\left(-\frac{\Delta t_{ij}}{T}\right)\cdot \left(1-\theta \cdot \frac{\max\left({UHI}_{i},{UHI}_{j}\right)}{\min\left({UHI}_{i},{UHI}_{j}\right)}\right)$$ (3)

${UHI}_{i}$ and ${UHI}_{j}$ represent the health index of city *i* and *j*; $d_{ij}^{\gamma }$ denotes the spatial distance between cities; ${\Delta t}_{ij}$ represents the travel time distance; c is the gravitational constant (set to 1), γ = 1.8 is the distance decay coefficient, and T is the time decay threshold. This model overcomes the limitations of traditional gravity models that only consider spatial distance by introducing the time dimension and an exponential decay function, making it more aligned with real-world collaborative mechanisms. This study built on the research of Wei et al. [32] and introduced $1-\theta \cdot \frac{\max\left({UHI}_{i},{UHI}_{j}\right)}{\min\left({UHI}_{i},{UHI}_{j}\right)}$ as the resource complementarity adjustment factor, where: $\frac{\max\left({UHI}_{i},{UHI}_{j}\right)}{\min\left({UHI}_{i},{UHI}_{j}\right)}$ reflects the similarity in health levels between two cities; when the *UHI* of two cities is similar (e.g., Beijing and Tianjin, both with relatively high *UHI*), this ratio approaches 1, and the adjustment factor tends toward 0, suppressing the gravitational value; when the UHI difference between two cities is significant, this ratio approaches 0, and the adjustment factor tends toward 1, leaving the gravitational value unsuppressed. θ is the competition coefficient, representing the weakening effect of resource similarity on collaborative gravity, and has a value of 0.3.

The parameters in the gravity model were determined as follows: The distance decay coefficient (γ = 1.8) falls within the range commonly used in spatial interaction models in regional studies (e.g., 1.5–2.0) and was finalized through iterative testing to best fit the observed inter-city patient flow data from a subset of the study regions. The competition coefficient (θ = 0.3) was set to reflect the moderate level of resource competition and functional similarity observed among Chinese cities of a similar administrative rank. The time decay threshold (T) was set to 2 h, which is a common tolerance for inter-city medical travel. The robustness of these parameter choices was subsequently confirmed through the sensitivity analysis presented in the “Model Validation” section.

#### Urban Health Network Model

Using the Pearson correlation coefficient and social network analysis (SNA), a dynamic collaborative network was constructed, calculating the Pearson correlation coefficient of gravitational values between cities $r_{ij}$ , with the formula:

$$r_{ij}=\frac{\sum_{t=1}^{T}\left(G_{ij}^{t}-\bar{G_{i}}\right)\left(G_{ji}^{t}-\bar{G_{j}}\right)}{\sqrt{\sum_{t=1}^{T}{\left(G_{ij}^{t}-\bar{G_{i}}\right)}^{2}\sum_{t=1}^{T}{\left(G_{ji}^{t}-\bar{G_{j}}\right)}^{2}}}$$ (4)

Among them, represents the gravitational value of city *i* to *j* at time *t*; $\bar{G_{i}}$ , $\bar{G_{j}}$ is the mean value. Subsequently, degree centrality was calculated to reveal the structural characteristics of the network, with the degree centrality formula as follows: $C_{D}\left(i\right)=\sum_{j=1}^{n}g_{ij}/n‐1$ . Here, $g_{ij}$ denotes the connection strength between city *i* and *j* (with values of 0 or 1).

### Model Validation and Robustness Analysis

This study employed the leave-one-out cross-validation (LOOCV) method to verify model stability. Specifically, each time one urban agglomeration dataset was excluded, the model was trained with the remaining three urban agglomeration datasets. The excluded urban agglomeration’s collaborative gravity value was predicted, and the root mean square error (RMSE) and coefficient of determination ( $R^{2}$ ) were calculated. The results show that the RMSE values were all less than 0.05 and that the $R^{2}$ values were all greater than 0.92, indicating that the model has good generalization capability. This study’s sensitivity analysis examined the variation in gravity values when adjusting the distance decay coefficient (1.5–2.0) and the time threshold T (±20%). The results also demonstrate that, when the distance decay coefficient varies by ±0.1, the gravity value fluctuates by less than 3%, and when the coefficient varies by ±20%, the fluctuation is less than 5%, indicating that the model is not sensitive to parameter changes and exhibits strong robustness.

## Results

### Regional Health Development and Collaborative Level

**FIGURE 1:**
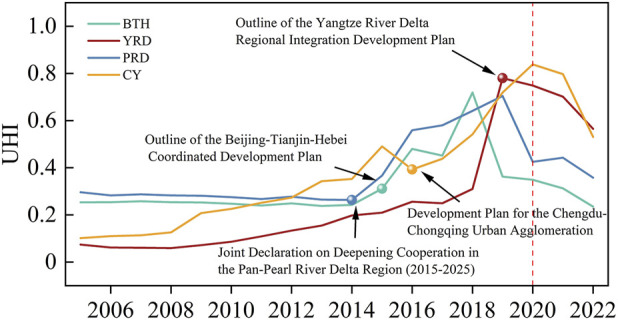
Changes in UHI across the studied urban agglomerations (Beijing-Tianjin-Hebei, Yangtze River Delta, Pearl River Delta region, and Chengdu-Chongqing, China, 2023) and the spatiotemporal variation and synergy index of urban agglomeration health development levels.

From Figure 1-UHI, it can be observed that the four urban clusters exhibit similar trends in public health levels, showing a generally upward trajectory before 2020, followed by a significant decline after 2020 due to the impact of a major public health emergency. Figure 1-HCDI reveals that from 2015 to 2019, the four study regions experienced a notable upward trend in their Regional HCDI. This trend correlates with the introduction of collaborative development policies in China during this period, such as the Beijing-Tianjin-Hebei Collaborative Development Plan Outline in 2015, the Yangtze River Delta Regional Integration Development Plan Outline in 2019, the Pan-Pearl River Delta Regional Deepening Cooperation Joint Declaration (2015–2025) signed in 2014, and the Chengdu-Chongqing Urban Cluster Development Plan in April 2016. Following the implementation of these regional collaborative policies under strong national-level coordination, the public health collaboration indices of the four major urban clusters saw substantial growth compared to previous years. This demonstrates that national policy support has played a powerful role in advancing public health development within specific regions.

### Network Structure of Collaborative Health Development in Urban Agglomerations

**FIGURE 2:**
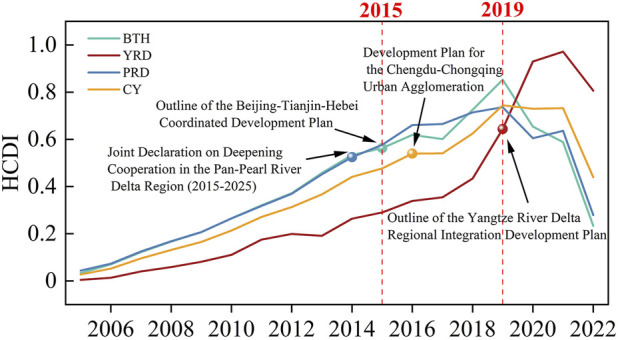
The Status of Health Coordination in Four Urban Agglomerations (Beijing-Tianjin-Hebei, Yangtze River Delta, Pearl River Delta region, and Chengdu-Chongqing region, China, 2023).

Using ArcGIS Map and Origin 2024 software, the previously constructed health collaboration gravity model was used to calculate the results and visualize the urban network, as shown in Figure 2. The importance of urban nodes in the region is represented by different colors and sizes; the thickness of the gravity lines indicates the intensity of collaborative gravity between cities, and the gradient from dark to light red represents a city’s health level. The overall spatial distribution of urban clusters reflects the development status of health initiatives among cities within the region, alongside the collaborative health development before and after the implementation of coordinated policies. It can be observed that, both before and after policy implementation, the UHI index of key cities within the clusters is higher than that of other cities in the region, indicating that the public health development in these key cities has consistently led the region. The strengthening of this radiating influence is corroborated by the steady rise in the overall HCDI for all urban agglomerations following the enactment of key regional policies around 2015–2016, as clearly depicted in Figure 2. This aggregate trend provides indirect evidence of intensifying inter-city connections, which is manifested in the network models as an enhanced gravitational pull from core cities.

However, it is also important not to overlook the role of geographical distance in regional health collaboration. The collaborative gravity between the majority of cities tends to exhibit lower intensity levels when the distance between them is large, whereas relatively closer cities—especially key cities and other cities in the region—show higher collaborative intensity. Examples include Beijing and Langfang.

Within urban clusters, higher traffic accessibility between cities corresponds to higher collaborative intensity levels in the gravity lines. Additionally, in terms of temporal evolution, the gravitational pull among cities in urban clusters has continuously strengthened over time, indicating a gradual increase in regional healthcare collaboration. Compared to the 2014–2018 period, medical resource collaboration intensity grew in a more pronounced way across all urban clusters during 2018–2023, demonstrating the rapid development of inter-cluster medical collaboration and the further enhancement of interactivity and synergy of medical resources during this period.

### Spatial Characteristics of Coordinated Health Development in Urban Clusters

**FIGURE 3:**
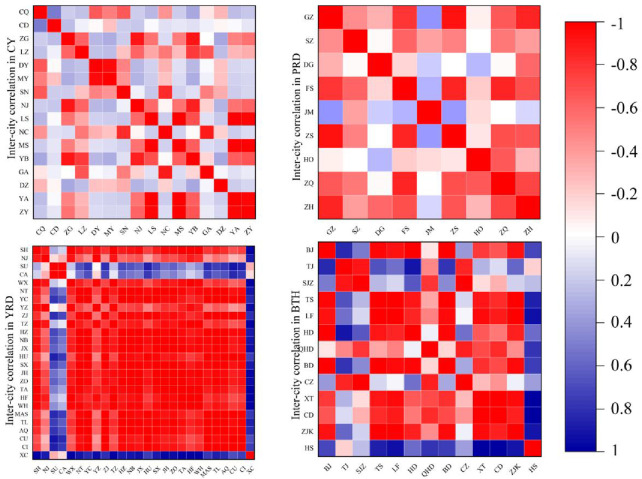
Heat maps of correlation for Beijing-Tianjin-Hebei, Yangtze River Delta, Pearl River Delta, and Chengdu-Chongqing (China, 2023).

**FIGURE 4:**
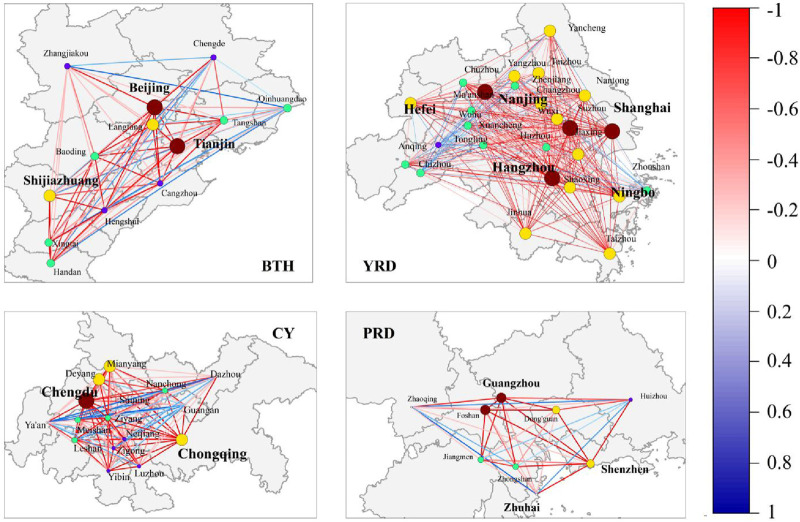
The collaborative structure (Beijing-Tianjin-Hebei, Yangtze River Delta, Pearl River Delta, and Chengdu-Chongqing, China, 2023).

Combining the previously calculated inter-city $r_{ij}$ and plotting Figure 3, this more intuitively reveals the public health collaborative development relationships within each study area—especially between non-core cities in the region. Figure 3 displays the correlation of inter-city health collaboration gravity values ( $G_{ij}$ ) across different urban clusters. Red indicates a positive correlation, with darker red representing higher values, while blue indicates a negative correlation, with darker blue representing lower values. The color intensity reflects the magnitude of the gravity value $G_{ij}$ and the strength of the correlation. From the inter-city correlation characteristics, the YRD exhibited the strongest synergy with a high-concentration positive correlation network across the entire region, while the CY, with its mixed red and blue blocks and low synergy among core city pairs, emerged as the region with the weakest synergy. Figure 4 was generated by integrating Figures 2, 3, illustrating the internal public health collaborative development structures of different urban clusters.

In Figures 3, 4, the analysis reveals the collaborative development structures and characteristics of the four major urban clusters between 2014 and 2023, as detailed below:

#### The Beijing-Tianjin-Hebei (BTH) Region Was Found to Exhibit a Unipolar Radiating Collaborative Structure

Beijing forms strong synergies (dark red) with neighboring cities, such as Langfang, Baoding, and Zhangjiakou, as evidenced by the thickest gravity lines radiating from Beijing in Figure 2, while cities farther from Beijing in the region also maintain notable connections (light red). This indicates high gravitational values between Beijing and regional cities, forming a strong correlation axis centered on Beijing, reflecting the spillover effects of the capital’s resources. Meanwhile, Tianjin, another key city in the region, is marginalized. Its collaboration with surrounding cities, such as Tangshan and Cangzhou, is weak (blue) due to functional overlap with Beijing, which suppresses its influence over neighboring cities. As the provincial capital of Hebei, Shijiazhuang faces similar challenges to Tianjin, compounded by its significantly lower economic level compared to Beijing and Tianjin. Additionally, due to industrial homogenization and internal competition among Hebei’s cities, Shijiazhuang lacks coordination, resulting in a collaborative vacuum with eastern Hebei and a pronounced spatial differentiation of “strong around Beijing.” Overall, the BTH region was found to present a pattern of “one dominant core, weak secondary cores, and fragmented peripheries.”

#### The Yangtze River Delta (YRD) Region Was Found to Exhibit a Polycentric Networked Collaborative Structure

The deep red coloration in the YRD indicates a high overall level of collaboration. Within the region, a strong tri-core collaborative network formed between SH-NJ-HZ, which are connected by the most robust gravitational links in the entire network visualization (Figure 2), with core cities demonstrating significant radiation effects on neighboring areas. Intra-provincial collaboration outperforms cross-provincial cooperation, while distant cities show weaker coordination. Centered on Shanghai, a deep red triangular zone emerges with Nanjing and Hangzhou. Among neighboring cities, Suzhou and Shanghai (100 km apart) appear deep red (high gravitational value), while cities such as Chizhou (over 300 km away) appear light blue. Intra-provincial city pairs, such as SU-WX, display red coloration, indicating strong collaboration. This demonstrates that in the YRD region, beyond traditional major cities, economically robust cities can also become focal points for regional public health collaboration.

#### The Pearl River Delta Region (PRD) Was Found to Exhibit a Core-Periphery Collaborative Structure

Guangzhou and Shenzhen form ultra-strong collaborative nodes as dual cores, with the gravitational connection between them being the strongest in the entire study, as represented by the thickest, darkest red link in Figure 2. SZ-DG and GZ-FS show deep red, while neighboring cities display a “core-periphery” gradient radiation structure due to geographical proximity. Collaboration among other cities within the same province is active, but the collaborative gap between distant cities and core areas is significant. Meanwhile, distant cities, such as Zhaoqing and Jiangmen, display noticeably lighter colors, indicating weaker collaboration with core cities.

#### The Chengdu-Chongqing Region (CY) Was Found to Exhibit a Dual-Core, Segmented, Policy-Mandated Collaborative Structure

Compared to other city clusters, CY appears lighter with more blue tones in Figure 3, indicating weaker regional collaboration. Collaboration between Chengdu and Chongqing is weak. While Chengdu shows localized red areas with policy-mandated neighboring cities, such as Ya’an, in the correlation heatmap (Figure 3), market-driven collaboration with cities, such as Ziyang, is negligible (appearing light blue/white in Figure 3). Non-core cities generally appear blue, showing an obvious lack of collaboration. The grid colors between Chengdu and Chongqing are notably lighter than those between other regional core city pairs. Secondary cities in Sichuan, such as Nanchong and Luzhou, appear blue, showing almost no collaborative signals.

## Discussion

This study provides the first systematic spatiotemporal analysis of health collaboration patterns across China’s four major urban agglomerations, revealing the distinct structural characteristics of their collaborative networks. Our findings contribute to the understanding of regional health governance in several important ways.

### Conclusion

Based on the above research findings, this article summarizes some correlations between regional public health collaborative development and key regional cities that may influence the study of coordinated regional public health development.

The PRD urban agglomeration exhibits a relatively high level of coordinated public health development, while the YRD urban agglomeration demonstrates more balanced public health coordination. Beijing and Chengdu exert significant influence over public health coordination within their respective urban clusters. The coordinated development of public health in the YRD region exhibits a multi-point pattern, while other regions show a trend of concentration around key cities. Collaborative relationships in health and wellness among cities of different scales exhibit distinct structures.

### Comparison With Existing Literature

Our identification of four distinct collaboration structures aligns with but also extends previous research on urban networks. The unipolar structure in BTH reflects the “core-periphery” patterns observed in previous studies of regional development in China [13], but our analysis reveals the specific mechanisms through which political centrality reinforces health resource concentration. The polycentric network in YRD demonstrates how economic integration can foster more balanced health collaboration, supporting the findings of regional integration studies in Europe and North America [21].

The core-periphery structure in PRD shows similarities to metropolitan governance patterns observed in global city-regions, where economic complementarity drives functional integration [37]. However, the dual-core, segmented structure in CY represents a unique pattern that reflects China’s specific regional development policies and the challenges of cross-provincial coordination.

### Methodological Contributions

Our improved gravity model, which incorporates both spatial and temporal dimensions along with a competition coefficient, addresses the limitations of traditional gravity models in health collaboration research. This approach provides a more nuanced understanding of how geographic proximity, temporal accessibility, and resource complementarity jointly shape collaboration patterns. The integration of policy quantification into our index system also represents an advancement in the measurement of the impact of governance factors on health collaboration.

### Policy Implications

The varying collaboration structures identified in our study suggest the need for differentiated regional health governance approaches. In unipolar structures, such as BTH, policies should focus on strengthening secondary nodes and reducing dependency on the core. In polycentric networks, such as YRD, the emphasis should be on enhancing connectivity between multiple centers. For core-periphery structures, such as PRD, policies should address the collaboration gaps between the core and peripheral areas. In dual-core segmented structures, such as CY, the priority should be to foster cross-jurisdictional integration and reduce policy fragmentation.

### Limitations and Future Research

This study has several limitations that should be addressed in future research. First, our indicator system, while comprehensive, may not capture all dimensions of urban health collaboration. Future studies could incorporate additional indicators, such as environmental factors, health outcomes, and social determinants of health. Second, our data ends in 2023, missing potential post-COVID-19 trends in health collaboration. Further research on post-COVID-19 development trends requires a longer time horizon to fully capture relevant dynamics. Third, the selection of economically developed cities within each agglomeration may introduce sampling bias.

Future research could expand the geographical scope to include more urban agglomerations, incorporate dynamic network analysis to track collaboration evolution in real time, and employ mixed-methods approaches to better understand the mechanisms underlying the observed collaboration patterns.
